# The New Face of Berries: A Review of Their Antiviral Proprieties

**DOI:** 10.3390/foods11010102

**Published:** 2021-12-31

**Authors:** Charlie Bernier, Coralie Goetz, Eric Jubinville, Julie Jean

**Affiliations:** Department of Food Sciences, Institute of Nutrition and Functional Foods (INAF), Université Laval, Quebec, QC G1V 0A6, Canada; charlie.bernier.1@ulaval.ca (C.B.); coralie.goetz.1@ulaval.ca (C.G.); eric.jubinville.1@ulaval.ca (E.J.)

**Keywords:** antiviral, berries, natural compound, inactivation, viral inactivation

## Abstract

Due to rising consumer preference for natural remedies, the search for natural antiviral agents has accelerated considerably in recent years. Among the natural sources of compounds with potential antiviral proprieties, berries are interesting candidates, due to their association with health-promoting properties, including antioxidant, antimutagenic, anticancer, antimicrobial, anti-inflammatory, and neuroprotective properties. The past two decades have witnessed a flurry of new findings. Studies suggest promising antiviral proprieties against enveloped and non-enveloped viruses, particularly of cranberries, blueberries, blackcurrants, black raspberries, and pomegranates. The aim of this review is to assemble these findings, to list the implied mechanisms of action, and thereby point out promising subjects for research in this field, in the hope that compounds obtainable from natural sources such as berries may be used someday to treat, or even prevent, viral infections.

## 1. Introduction

Fruits and vegetables are part of a healthy diet, and their daily consumption is strongly recommended. Whereas the variety of fruits and vegetables available to consumers is vast, some of them, such as berries, have particularly interesting properties [1]. Berries differ from larger fruits by containing a broader range of bioactive components. In recent decades, these compounds have been shown to possess interesting proprieties including antioxidant, antimutagenic, anticancer, antimicrobial, anti-inflammatory, and neuroprotective properties, described in three reviews [2,3,4].

Among these properties, one of the most studied has been the antimicrobial activity attributed to cranberries, and its impact on the incidence of urinary tract infections. Cranberry polyphenols have been shown to interfere with bacterial adhesion to the urinary tract, and thereby prevent colonization and progressive infection [5]. This discovery is important since urinary tract infection is now the second most common type of bacterial infection worldwide, but also because of the increasing prevalence of antibiotic resistance among bacterial pathogens [5,6].

Antioxidant proprieties are also increasingly sought since they are widely believed to protect the body against diseases that are often associated with oxidation by free radicals. The antioxidant capacity of berries is relatively high among fruits and vegetables, due to the presence of vitamins, anthocyanins, catechins, ellagic acid, gallic acid, quercetin, and various related compounds at considerable concentrations [7,8,9,10,11,12].

A lesser-known property of berries is their antiparasitic activity. Blueberry extract appears to cause spontaneous excystation of *Giardia duodenalis* and *Cryptosporidium parvum* [13]. The process of excystation is normally under strict control, and if it occurs suddenly, the parasite emerging from its cyst is unlikely to be viable [14]. *Giardia duodenalis* in its trophozoite stage can be killed in vitro by contact with a polyphenol extract of cloudberry containing only 10 to 20 μg of gallic acid equivalent per milliliter [15]. Since cloudberries typically contain of about 150 mg of phenolic compound per 100 g, enough of the active compounds could reach the small intestine to be effective [16,17]. The efficacy of cloudberry has been found comparable to that of the antiprotozoal drug metronidazole [18].

Antimutagenic, anticancer, anti-inflammatory, and neuroprotective properties of berries have also been studied widely, and are at least mentioned in review articles [2,3,4]. In contrast, the antiviral activity of berries has received attention for little more than a decade, but has not been reviewed for viruses in general, or for any broad range thereof. Meanwhile, antiviral research has been a major field for decades, its importance having grown considerably since the spread of human immunodeficiency virus in the 1980s [19]. Recent events regarding the coronavirus pandemic have of course further heightened the urgency of research in this field.

In the search for the ideal antiviral agent, natural compounds have been investigated, due largely to their long history of use in traditional medicine. Written records of the use of plants for medicinal purposes in Mesopotamia date as far back as 2600 BCE [20]. The current search for antiviral substances of natural origin initially focused to a large extent on herbs and spices before broadening to include marine-derived products. This led to the identification of some active compounds in traditional foods, such as algae [19,21,22]. Among the most active of these were flavonoids. It was thus inevitable that the antiviral potential of berries would be tested, since they are rich in flavonoids [23]. The studies summarized below describe the wide range of promising antiviral proprieties that have been attributed to berries against both enveloped and non-enveloped viruses. These proprieties depend strongly on the composition of the berry tested, and on the type of virus targeted. The variety of phytochemicals in berries makes them very interesting as sources of antiviral compounds, since they are almost as diverse as the viruses, thereby increasing the likelihood of finding a match. This review aims to present the current results from published articles regarding the potential ability of berries to inactivate different viruses. The viruses chosen for this review are viruses that are of great concerns in the population worldwide because they are currently known to cause infections and/or to be carried by a high percentage of the population.

## 2. Antiviral Mechanism

**Figure 1:**
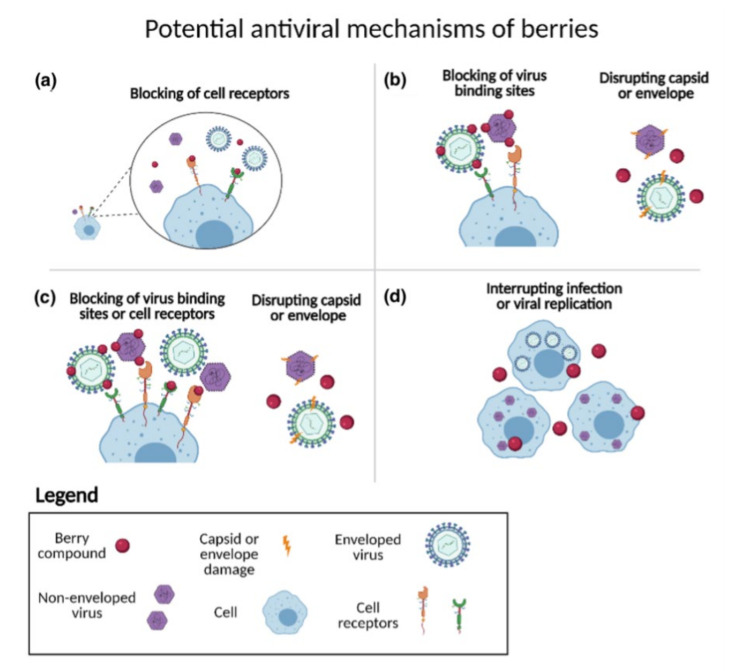
Graphical description: (**a**) pre-incubation of the cells, (**b**) pre-incubation of the virus, (**c**) co-incubation, (**d**) post-incubation.

Depending on how a berry is tested as a source of compounds that inhibit viral propagation, a different mechanism of antiviral action may be suggested by the results. The four most common in vitro testing methods are described above (Figure 1).

### 2.1. Pre-Incubation of the Host Cells

This method comprises incubating the culture of mammalian host cells with a solution of extract from the berry for pre-determined exposure times, after which, the solution is removed and replaced by various dilutions of virus in suspension to allow infection to occur [24]. Since under these conditions only the cultured cells have been in contact with the compounds extracted from the berry, failure of the virus to replicate is due necessarily to a mechanism associated with the cells. One or more compounds could bind to cell surface receptor molecules, and thereby block the specific sites to which the virus must attach to invade the cell, and replicate [25].

### 2.2. Pre-Incubation of the Virus

This method comprises incubating the virus in the tested berry extract solution for pre-determined times, then recovering the virus, and resuspending it at various dilutions for contact with the host cell culture [24]. If the virus fails to replicate under these conditions, the antiviral mechanism is associated with the virus itself, for example, one or more berry compounds binding to one or more molecules on the virion surface, and either masking the structure that the virus uses to attach to host cells, or possibly disrupting the viral envelope or capsid, and thereby inactivating the virion permanently [24,26].

### 2.3. Co-Incubation

In this method, the berry extract solution and the virus at suitable dilutions are both in contact with the host cell culture throughout the incubation period, and, hence, during each stage of any infection that occurs [24]. If the virus fails to replicate under these conditions, the antiviral mechanism occurs during the process of infection. This test by itself does not indicate whether the berry compound or compounds involved bind to the virions or to the cells, or if they interfere with some physicochemical or enzymatic process that the virus requires in order to gain entry into the host cells [24,27]. However, if the other two tests for antiviral activity are negative, a positive co-incubation test result suggests interference with a process other than attachment.

### 2.4. Post-Incubation

This method comprises incubating a culture of already infected host cells with berry extract solution. Under these conditions, if infection is halted relative to an infected control culture, one or more berry compounds must be getting inside the host cells, and somehow interfering with the process of viral replication [24].

## 3. Effect of Berries on Enveloped Viruses

Enveloped viruses are characterized by a lipid membrane surrounding the capsid. In order to infect a host, these viruses must fuse with the cell membrane, which is mediated by a viral fusion protein [28]. Mechanisms by which berry compounds are believed to inhibit or prevent infection by enveloped viruses are listed in Table 1, and are described below.

### 3.1. Hepatitis C Virus

The hepatitis C virus (HCV) is an RNA virus classified in the genus *Hepacivirus* in the family *Flaviviridae* [29]. Infections by HCV are the main cause of chronic liver disease and hepatocellular carcinoma [30]. Even though these infections are now much better controlled thanks to advances in virology and diagnostics, no anti-HCV vaccine is yet available [31,32]. Most new infections occur in developing countries, where they are a major burden on national economies and healthcare systems [31]. According to the World Health Organization (WHO), 71 million people currently live with chronic HCV infection [33]. Dose-dependent suppression of HCV sub-genome expression in a replicon cell system exposed to proanthocyanidin extract from blueberry leaves has been observed in an older well-designed study, but the mechanism of this activity was unclear, and remains to be identified [34]. Then, a study more focused on the mechanism presents another type of inhibition: the polyphenolic anthocyanidin delphinidin, which is the compound responsible for the bluish-purple pigment in berries, inhibits HCV adhesion when cells and virus are co-incubated in its presence and the virus has been pre-incubated with it. Post-incubation exposure of HCV-infected cells to delphinidin appears not to affect viral replication [24]. This suggests that it interferes with viral entry into cells rather than with viral replication. According to this article, polyphenols found in delphinidin seem to be able to change the shape of the viral envelope, and render the E1E2 envelope glycoprotein unable to interact with the cell surface [24]. However, regarding the pathway for HCV initiation and viral infection of host cells well presented by Zeisel and al., there could also be a disruption of other important entry factors such as Apolipoprotein E, an essential particle in the infectivity of HCV [35]. These results suggest promising avenues to investigate for preventing HCV infection, since the inhibitory actions are occurring in the very first steps of infection, thus preventing infection.

### 3.2. Herpes Simplex Virus

The herpes simplex virus (HSV) is classified in the family *Herpesviridae* and genus *Simplexvirus*, which are double-stranded DNA viruses. Herpes simplex virus types 1 (HSV-1) and type 2 (HSV-2) are the two more related of the three viruses that belong to the a-*Herpesvirinae* subfamily [36]. Both are known to cause latent infections in humans, often producing blisters at the site of primary infection [37]. A study has shown a major reduction in HSV-1 replication in the presence of low concentrations of blackberry extract, and reductions to undetectable levels at higher concentrations, especially when the virus was pre-incubated with the extract [38]. The antiviral mechanisms appear to include interference with virion adsorption to the host cell, and disruption of one or more intracellular steps in the viral replication process [38]. Another berry that appears to contain compounds that could block HSV-1 infection is the blackcurrant. Anti-adsorption and anti-replication activities have been observed [39]. Cranberry extract containing a high concentration of proanthocyanidins was next to be identified as a potential anti-HSV-1 and anti-HSV-2 substance, based on cell pre-incubation tests, suggesting that viral attachment to cells was inhibited [25]. This effect was later found to be concentration-dependent. Immunoblotting was used to demonstrate that the extract inhibits the expression of specific HSV proteins, which led to more studies of cranberry extract in the hope of finding an efficacious inhibitor of HSV [25].

### 3.3. Influenza Virus

The *Orthomyxoviridae* family currently contains four types of influenza virus: A, B, C, and D. Only influenza types A, B, and C infect humans [40,41,42,43]. These viruses are known worldwide as the cause of seasonal respiratory illness or flu [44]. The WHO estimates the number of influenza infections per year at up to 1 billion during the past decade, and the resulting deaths at 500,000 per year [45]. The most common symptoms are fever, cough, and nasal congestion [44]. In one study, elderberry extract was shown to inactivate influenza A (H1N1) in a dose-dependent manner [46]. Flavonoids in the extract were tested in direct binding assays, and shown to bind to the viral envelope and, more precisely, to a hemagglutinin domain. This could inhibit infection by blocking the ability of H1N1 to bind to host mucous membrane cells by attaching itself to the hemagglutinin domain, since the more particles there are, the less infection occurs [46]. Antiviral activity of blackcurrants against influenza A virus has also been studied. Blackcurrant extract was found to inhibit viral adsorption to cultured cells by more than 95% [39]. This effect was observed at acidic pH and at neutral pH. The antiviral mechanism was not identified by the authors, and remains unknown [39]. However, regarding the viral fusion section presented by Dou et al. to fuse with the cells, the virus must go through many steps and conformational changes where the extract could potentially interfere. The first and main one is the cleavage of the virus by the cells protease to expose his fusion peptide. Without this fusion peptide, the virus could not fuse properly [47]. An inhibition at this stage would prevent infection, and could only be detected when the extract is present during the infection stage. This could be a potential mechanism to further experiment. The relationship between the polyphenol content of berries and their anti-influenza activity has later been studied [48]. It has been suggested that berry polyphenols inhibit adsorption of influenza A virus to cells likely at the attachment point of the virus [48]; hence, potentially interfering with the HA receptor-binding site [47]. In a later study of the antiviral activity of blackcurrant extract against influenza A and B, it was found that a shorter contact time with the extract was equally effective [49]. They suggested the hypothesis that blackcurrant compounds affect the cell-surface, the viral receptor, or possibly the virus hemagglutinin protein [49]. Later that year, cranberry phytochemicals were studied as a potential enhancer of γδ-T cell proliferation. These cells are the first line of defense against respiratory viruses, and an increase in their numbers could therefore help reduce the symptoms caused by influenza once infection has occurred [50]. In a clinical trial, daily consumption of a cranberry beverage for 10 weeks was associated with a 5-fold increase in γδ-T cell proliferation, and with attenuated symptoms of influenza-like fever, cough, and headache [50]. In a later in vitro study of cranberry as an inhibitor of influenza A and B, an extract containing a high concentration of proanthocyanidins inhibited both viruses in a concentration-dependent manner [27]. The extract was more effective in pre-infection or co-infection than in post-infection contact, suggesting that its impact is more on viral adsorption, and that it does indeed interfere with hemagglutinin. Based on these results, cranberry extract could have a role to play in lessening the prevalence or the severity of influenza infections [27]. Regarding all the above studies, berries have an important role to play in the medical field. They could help prevent infections, and even when the infection has already occurred, they could help lessen the symptoms.

### 3.4. Respiratory Syncytial Virus

The respiratory syncytial virus (RSV) is a single-stranded RNA virus of the genus *Pneumovirus* in the family *Paramyxoviridae* [51]. This highly contagious virus affects mainly infants under the age of two. The symptoms are usually mild, but can be severe and even life-threatening [51]. In industrialized countries, this virus rarely causes death, unlike in developing countries, where it is more problematic [52]. In a study of the antiviral properties of blackcurrant extract, RSV adsorption to the host cell surface and viral replication were both inhibited [39]. Acidic pH favored the inhibition of replication, whereas adsorption was inhibited regardless of pH, suggesting that the viral molecules involved in each of these steps have different physicochemical sensitivities. Also, regarding Battles et al.’s review and, more precisely, the adsorption part, it could either interfere during the attachment or during the membrane fusion [53]. For the attachment, it is regulated by a G protein that attaches the virions to the cell surface by interactions with the cell’s attachment factors. This process involves binding to disaccharides polymers, named glycosaminoglycans, that are present on the cell’s surface [53]. An alteration of any of these sites could result in an inhibition of infection. For the fusion part, it is less known how it happens, but it is believed that it might be pH dependent, or that it requires a low pH to happen, and since the pH did not affect the adsorption part, it is most likely that the viral inhibition happens at the attachment part rather than the fusion part [53]. These results warrant further investigation, especially for the understanding of the replication part, since there is still no effective antiviral agent to treat RSV infection [39].

**Table 1:** Summary of reported antiviral effects of berries or constituents thereof on enveloped viruses.

Virus	Berry	Component	Concentration ^1^	Exposure (min or h)	Temp.	Viral Reduction ^1^	Ref.
Hepatitis C virus	Various	Delphinidin	3.7 μM	2 h	37 °C	50%	[24]
Herpes simplex 1	Blackberry	Extract	≥56 μg/mL	N/A	N/A	>99% (replication)	[38]
	Blackcurrant	Extract	0.5%	N/A	N/A	50% (replication)	[39]
	Blackcurrant	Extract	10%	10 min	N/A	>95% (adsorption)	[39]
	Cranberry	Extract	14.2 μg/mL	1 h	37 °C	50% (replication)	[25]
Herpes simplex 2	Cranberry	Extract	9.6 μg/mL	1 h	37 °C	By half (replication)	[25]
Influenza	Cranberry	Juice	Not specified	In vivo	In vivo	Symptoms attenuated	[50]
Influenza A	Elderberry	Extract	1108 μg/mL	1 h	23 °C	95%	[46]
	Blackcurrant	Extract	10%	10 min	N/A	>95% (adsorption)	[39]
	Blackcurrant	Extract	10%	5 min	37 °C	Undetectable titer (adsorption)	[49]
	Cranberry	Extract	4.7 μg/mL	1–2 h	37 °C	50%	[27]
Influenza B	Blackcurrant	Extract	10%	10 min	N/A	>95% (adsorption)	[39]
	Blackcurrant	Extract	10%	5 min	37 °C	Undetectable titer (adsorption)	[49]
	Cranberry	Extract	4.2 μg/mL	1–2 h	37 °C	50%	[27]
Respiratory syncytial virus	Blackcurrant	Extract	10%	10 min	N/A	>95% (adsorption)	[39]

^1^ The numbers reported do not take the margin of error into consideration, N/A equal not available.

## 4. Effect of Berries on Non-Enveloped Viruses

Non-enveloped or “naked” viruses do not have a lipid membrane. They are usually more resistant than enveloped viruses to physicochemical perturbations, solvents, and extreme temperature [54]. A non-enveloped virus remains infectious if its capsid is intact, and host-cell receptors are exposed [55]. Antiviral effects of berry extracts on non-enveloped viruses are listed in Table 2, and described below.

### 4.1. Adenovirus

Adenoviruses are double-stranded DNA viruses classified in the genus *Mastadenovirus* in the *Adenoviridae* family [56,57]. They are known to affect the respiratory tract, and to infect mainly children, who lack humoral immunity [58,59]. Susceptibility in adults is mostly due to immunocompromised condition [58]. A 2012 study of the antiviral effects of blackcurrant extract showed that adenovirus propagation was inhibited, in part, at the adsorption stage [39]. The exact mechanism remains unknown, but the effect is less apparent at pH near neutrality, suggesting that raising the pH reduces the concentration of one or more active compounds in their free forms by causing the formation of insoluble precipitates [39]. These results are interesting, since they suggest that viral adsorption is mediated by specific molecules that are affected by the pH, or that the extract can be mediated by the pH.

### 4.2. Aichi Virus

Aichi viruses are single-stranded RNA viruses with an icosahedral morphology [60]. They belong to the genus *Kobuvirus* in the *Picornaviridae* family [60], the members of which are around 30 nm in diameter, which is very small [61]. The Aichi virus is currently considered to be a causative of viral gastroenteritis with fever, diarrhea, and nausea [61]. Its role in this illness remains unclear, since it appears to occur mostly in coinfection with other viruses [60]. Other studies have shown it to be the sole cause of many infections [60,62]. Many studies have obtained antiviral activities on Aichi virus. Among them, PACs, juice, polyphenols, and gallic acid from cranberries, pomegranates, and black raspberries were studied, and attributed antiviral proprieties, but no mechanisms were defined precisely [63,64,65,66,67]. However, one study has shown its time-dependent and concentration-dependent sensitivity to blueberry proanthocyanidins, which can reduce its plaque-forming titer to undetectable levels. Blueberry juice was also found to inhibit its plaque-forming titer to undetectable levels. Again, the antiviral effect appears to be more effective at acidic pH [63]. This study concluded that the proanthocyanidins must be present in their entirety to inactivate the Aichi virus, and that chemically modified forms are less effective. It also concluded that when diluted in apple juice, the blueberry proanthocyanidins had enhanced antiviral activity [63]. No specific mechanism or active compound will be identified without further study, since even the mechanism of infection of the Aichi virus is little known. The current information regarding its mechanism of infection refers to endocytosis mediated by receptors. With this information, the mechanism of inhibition would refer to a blockage of receptors from the cell or the virus [68].

### 4.3. Hepatitis A Virus

The hepatitis A virus (HAV) is a single-stranded RNA virus classified in the genus *Hepatovirus* in the family *Picornaviridae* [69]. It is routinely transmitted via the fecal–oral route, and is commonly known worldwide. Foodborne outbreaks of HAV illness occur frequently [70]. In recent years, it has become less prevalent in North America due to vaccination, but is still a concern in developing countries, where vaccination is expensive, and natural products are preferred medicines [70]. The WHO estimates that approximately 1.5 million people are infected each year worldwide [71]. Since HAV is a very resistant virus, few studies have been published to date on its inactivation by natural products. In the most notable study, infectious titers were reduced to below detectability by blueberry proanthocyanidins, again more certainly at acidic pH, at which these compounds are more stable [26]. Blueberry juice showed fewer antiviral proprieties. The juice and the extracted compounds were both cytotoxic to the cultured cells during long incubation time or high concentration, respectively. The antiviral effect could be due to the blocking of receptors on the cell membrane or of the viral ones, or possibly to disruption of the capsid. And again, the proanthocyanidin portion does not seem to inhibit by interfering with viral replication [26]. These results nevertheless warrant further study, given the potential appeal of berry-based prophylaxis in the populations most concerned by HAV.

### 4.4. Human Papilloma Virus

The human papilloma virus is a double-stranded DNA virus classified in the genus *Alphapapillomavirus* in the family *Papillomaviridae* [72]. It is known as the agent of one of the most prevalent viral diseases that is sexually transmitted. Its prevalence is high in the population, regardless of geographic location. In many cases, it causes only benign lesions, whereas in others, it can cause tumors and even cancer [73]. It is also known as the primary cause of cervical cancer [74]. A berry compound known as ellagic acid has been tested in a phase 1 clinical trial [75]. For this purpose, tablets were produced containing 16 mg of ellagic acid with 100 mg of pulp from the fruit of an evergreen tree called Cherimoya, soursop, custard apple, and other common names (*Annona muricata*). The participants consumed these tablets or a placebo once daily for several weeks. Cervical cytological (Pap) examination showed fewer abnormal test results in the ellagic acid/berry group, suggesting that the compound had an antiviral effect that could help to prevent infection, and possibly contribute to successful treatment of the infection [75]. The researchers concluded that the effect was most likely due to the antioxidant proprieties of the compounds. It is also possible that one or more antioxidant compounds also had the property of interfering with or even stopping the progression of cervical disease [75].

### 4.5. Norovirus

Norovirus is a genus of RNA virus within the *Caliciviridae* family. They are known mainly as human enteric pathogens, but some strains also affect animals [76]. Human norovirus was the first virus shown to cause gastroenteritis [77,78] Originally known as the Norwalk virus, human norovirus is one of the main viral causes of foodborne infections. It has been an emerging problem particularly in North America [79,80]. The effect of food extract and juices on its ability to bind to cultured cells has been tested. Cranberry, cranberry-pomegranate juice, and raspberry extract have been found at least somewhat effective. The juices appear to interfere with binding of the virus, and therefore could be helpful in the future for slowing foodborne transmission [81].

Since laboratory replication of human norovirus is difficult for the moment, murine norovirus (MNV-1) is used as a surrogate in assays of antiviral candidates [82]. Pomegranate polyphenols were one of the first berry components to reduce infectious titers of MNV-1 [64], along with cranberry juice and polyphenols [65]. Later experiments with blueberry juice also showed a sufficient antiviral effect to warrant testing under other experimental conditions, and with other types of berries [83]. Meanwhile, cranberry, grape, orange, and black raspberry juices were tested against MNV-1. Black raspberry juice was the most potent inhibitor of plaque formation in co-incubation experiments. The antiviral effect again seemed to occur during adsorption. The other juices did not have any inhibitory effects [66]. Blueberry juice and proanthocyanidins were later retested against MNV-1 in short exposure experiments. Inactivation by the concentrated compounds was complete, whereas juice had more effect under longer exposure [26]. Blocking of cell-membrane receptors was found to be the most likely mechanism of action [26]. In the case of black raspberry seed extract, the most favorable test condition was treatment of the cells in the presence of the virus. Among the polyphenolic compounds tested, catechin and ellagic acid (both abundant in black raspberry) showed respectively weak and negligible effects, whereas gallic acid, a minor component, showed an interesting reduction, along with cyanidin-3-glucoside, another minor component [67]. In another retest of blueberry proanthocyanidins, better results were obtained when the extract was diluted in apple juice. Apple juice by itself had no significant effect on MNV-1 titer, suggesting a symbiosis effect [84]. Additional research would no doubt lead to more potent extracts of berries, and hopefully to the understanding of the mechanism of action against noroviruses.

### 4.6. Rotavirus

Rotavirus is a double-stranded RNA virus classified in the genus *Rotavirus* in the family *Reoviridae* [85]. It is known worldwide as the main cause of gastroenteritis in children [86]. In developed countries, vaccination has helped reduce the number of cases, but rotavirus remains responsible for nearly 48,000 child deaths per year, mainly in developing countries, where people tend to rely on more accessible and more affordable natural medicinal products [87,88]. Cranberry juice appears to contain a potent inhibitor of the hemagglutination activity of rotavirus SA-11, one that binds to or otherwise alters the viral glycoprotein, and renders it incapable of attaching to its receptor cell surface molecule; although, alteration of the attachment site cannot be ruled out, since entry of the virus is mediated at the hemagglutination step [89]. In subsequent assays of the antiviral activity of cranberry and grape juices and their proanthocyanidins, the capsid integrity of the rotavirus was affected at acidic pH, but 50% less at pH 7.0, except for grape proanthocyanidins, which were equally effective in acidic and neutral media [90]. This difference was attributed to differences in the molecular structures of type A and type B proanthocyanidins [91].

**Table 2:** Summary of reported antiviral effects of berries or constituents thereof on non-enveloped viruses (or “naked” viruses).

Virus	Berry	Component	Concentration ^1^	Exposure	Temp.	Viral Reduction ^1^	Ref.
Adenovirus	Blackcurrant	Extract	10%	10 min	N/A	73% (adsorption)	[39]
Aichi virus	Blueberry	Juice	N/A	24 h	37 °C	Undetected	[63]
	Blueberry	PACs	2 mg/mL	6 h	37 °C	Undetected	[63]
	Blueberry	PACs	2 mg/mL *	30 min	37 °C	Undetected	[63]
	Blueberry	PACs	5 mg/mL	3 h	37 °C	Undetected	[63]
	Pomegranate	Polyphenols	4 mg/mL	1 h	23 °C	Undetected	[64]
	Pomegranate	Juice	N/A	1 h	23 °C	1.2 log_10_ PFU/mL	[64]
	Cranberry	PACs	0.3 mg/mL	1 h	23 °C	Undetected	[65]
	Cranberry	Juice	N/A	1 h	23 °C	Undetected	[65]
	Blueberry	Juice	N/A	24 h	4 °C	Undetected	[83]
	Black raspberry	Juice	3%	1 h	23 °C	100%	[66]
	Black raspberry	Gallic acid and C3G	100 μM	1 h	37 °C	65%	[67]
	Black raspberry	Seed extract	1 mg/mL	1 h	37 °C	84%	[67]
	Blueberry	Juice	N/A	3 h	37 °C	Undetected	[26]
	Blueberry	PACs	0.5 mg/mL	1 h	37 °C	Undetected	[26]
	Blueberry	PACs	1 mg/mL *	15 min	37 °C	Undetected	[84]
Hepatitis A virus	Blueberry	Juice	N/A	24 h	37 °C	1.86 log_10_ PFU/mL	[26]
	Blueberry	PACs	2 mg/mL	30 min	37 °C	Undetected	[26]
Human papilloma virus	Berries	Ellagic acid	16 mg/mL **	In vivo	In vivo	In abnormal Pap test results	[75]
Murine Norovirus	Pomegranate	Polyphenols	16 mg/mL	1 h	23 °C	72%	[64]
	Cranberry	Juice	N/A	1 h	23 °C	41%	[65]
	Cranberry	PACs	0.6 mg/mL	1 h	23 °C	59%	[65]
	Black raspberry	Juice	6%	1 h	N/A	96%	[66]
	Blueberry	Juice	N/A	21 days	4 °C	48%	[83]
	Black raspberry	Gallic acid and C3G	100 μM	1 h	37 °C	50%	[67]
	Black raspberry	Seed extract	1 mg/mL	1 h	37 °C	87%	[67]
	Blueberry	Juice	N/A	6 h	37 °C	Undetected	[26]
	Blueberry	PACs	1 mg/mL	3 h	37 °C	Undetected	[26]
	Blueberry	PACs	1 mg/mL *	15 min	37 °C	Undetected	[84]
Rotavirus	Cranberry	Juice	>20%	30 min	23 °C	Total inhibition of hemagglutination	[89]
	Cranberry	Juice	100%	5 min	23 °C	93% loss of capsid integrity	[90]

^1^ The numbers reported do not take the margin of error into consideration. N/A = not available, PACs = proanthocyanidins. * Diluted in apple juice; ** 100 mg of *Annona muricata* fruit pulp was also present.

## 5. Conclusions and Prospects

Most of the studies mentioned in this review suggest that berries contain substances that have some antiviral activity at least in vitro against a wide variety of enveloped and non-enveloped viruses. Only few papers presented a non-effective reduction of the viruses by berries [66]. The more recent studies continue to show this for a lengthening list of berries. In some cases, the activity can be improved by combining an extract of one origin with a carrier of another origin. The possibility of synergism among naturally occurring antiviral compounds needs to be investigated. However, since each virus is different, only research will allow identification of the compounds that underlie the antiviral effect, not to mention the mechanism of action. More conditions need to be tested before their future use in antiviral medicine can even be contemplated. We expect that in some cases, a single compound might be involved, which could be extracted and purified. Cranberry extract is a likely candidate, in view of its richness in flavonoids. In the shorter term, more or less purified extracts containing active molecules could be used in industries to reduce the viral load on the consumable products. Some cleaning wipes or washing liquids could be an option to clean the outside of the fruits. The antiviral mechanism implied in positive tests based on the cell culture pre-treatment method would be suitable for screening candidate products for this application. Another application would be treatment of viral infections for which no standard treatment yet exists. In this case, candidate screening could be based on pre-treatment of the virus or virus/cell co-treatment. Natural compounds from edible sources would be more accessible, especially in developing countries [70], and would likely be more readily accepted, since they would be perceived as non-toxic and unlikely to produce harmful side-effects [20]. The possibility of preventing and treating viral infections using natural products appears to be real, and research on the mechanisms of antiviral action of natural compounds found in foods such as berries should be encouraged.

## Data Availability

Not applicable.
